# A Longitudinal, Observational Study of Etiology and Long-Term Outcomes of Sepsis in Malawi Revealing the Key Role of Disseminated Tuberculosis

**DOI:** 10.1093/cid/ciab710

**Published:** 2021-08-18

**Authors:** Joseph M Lewis, Madlitso Mphasa, Lucy Keyala, Rachel Banda, Emma L Smith, Jackie Duggan, Tim Brooks, Matthew Catton, Jane Mallewa, Grace Katha, Stephen B Gordon, Brian Faragher, Melita A Gordon, Jamie Rylance, Nicholas A Feasey

**Affiliations:** 1 Malawi Liverpool Wellcome Programme, Blantyre, Malawi; 2 Department of Clinical Sciences, Liverpool School of Tropical Medicine, Liverpool, United Kingdom; 3 Department of Clinical Infection, Microbiology and Immunology, Institute of Infection, Veterinary and Ecological Sciences, University of Liverpool, Liverpool, United Kingdom; 4 Rare and Imported Pathogens Laboratory, Public Health England, United Kingdom; 5 College of Medicine, University of Malawi, Malawi

**Keywords:** Africa south of the Sahara, critical illness, tuberculosis, HIV, antimicrobial resistance

## Abstract

**Background:**

Sepsis protocols in sub-Saharan Africa are typically extrapolated from high-income settings, yet sepsis in sub-Saharan Africa is likely caused by distinct pathogens and may require novel treatment strategies. Data to guide such strategies are lacking. We aimed to define causes and modifiable factors associated with sepsis outcomes in Blantyre, Malawi, in order to inform the design of treatment strategies tailored to sub-Saharan Africa.

**Methods:**

We recruited 225 adults who met a sepsis case definition defined by fever and organ dysfunction in an observational cohort study at a single tertiary center. Etiology was defined using culture, antigen detection, serology, and polymerase chain reaction. The effect of treatment on 28-day outcomes was assessed using Bayesian logistic regression.

**Results:**

There were 143 of 213 (67%) participants living with human immunodeficiency virus (HIV). We identified a diagnosis in 145 of 225 (64%) participants, most commonly tuberculosis (TB; 34%) followed by invasive bacterial infections (17%), arboviral infections (13%), and malaria (9%). TB was associated with HIV infection, whereas malaria and arboviruses with the absence of HIV infection. Antituberculous chemotherapy was associated with survival (adjusted odds ratio for 28-day death, 0.17; 95% credible interval, 0.05–0.49 for receipt of antituberculous therapy). Of those with confirmed etiology, 83% received the broad-spectrum antibacterial ceftriaxone, but it would be expected to be active in only 24%.

**Conclusions:**

Sepsis in Blantyre, Malawi, is caused by a range of pathogens; the majority are not susceptible to the broad-spectrum antibacterials that most patients receive. HIV status is a key determinant of etiology. Novel antimicrobial strategies for sepsis tailored to sub-Saharan Africa, including consideration of empiric antituberculous therapy in individuals living with HIV, should be developed and trialed.

## Teaser

We describe etiology and long-term outcomes of sepsis in Malawi in order to inform urgently needed locally adapted sepsis protocols. Disseminated tuberculosis dominates, and receipt of antituberculous chemotherapy is associated with survival. Significant post-discharge mortality is driven by late deaths in people living with human immunodeficiency virus.

Sepsis, a life-threatening organ dysfunction triggered by a dysregulated host response to infection [1], is estimated to cause 11 million deaths worldwide each year, with a disproportionate burden on low- and middle-income countries (LMIC) including sub-Saharan Africa [2]. Progress in improving sepsis outcomes in high-income settings has been made through early recognition and timely delivery of basic care including rapid administration of appropriate antimicrobial therapy and fluid resuscitation [3]. In sub-Saharan Africa, however, mortality remains high [4].

Many aspects of optimal sepsis management are, in principle, deliverable in resource-limited hospitals. However, applying sepsis protocols derived from high-resource settings to hospitals in sub-Saharan Africa has resulted in unexpected results, the most well-known being the harmful effect of liberal intravenous fluid therapy [5, 6]. To develop LMIC-targeted sepsis protocols, data from LMIC are urgently needed. This was highlighted in the recent World Health Organization (WHO) global report on the epidemiology and burden of sepsis [7] in which sepsis etiology and long-term sequelae were identified as particular gaps.

Standard antimicrobial treatment for sepsis in both high- and low-resource settings typically consists of broad-spectrum antibacterial therapy. However, limited available data from sepsis [4, 8] and fever etiology [9] studies in sub-Saharan Africa suggest that mycobacterial, viral, bacterial zoonotic, and parasitic causes of illness are common and not covered by standard antibacterial therapy [10]. Data on sepsis etiology beyond bloodstream infection in sub-Saharan Africa are lacking [4]; however, aligning the causes of infection with their effective treatments is central to not only preventing death and disability but also reducing unnecessary antimicrobial use, a key driver of antimicrobial resistance.

To address these data gaps, we provide a description of sepsis etiology in Blantyre, Malawi, describe long-term sepsis outcomes, and identify elements of current sepsis management in sub-Saharan Africa that are associated with outcome.

## METHODS

### Study Setting and Design

We undertook an observational cohort study, recruiting at Queen Elizabeth Central Hospital, Blantyre, Malawi, a 1300-bed government teaching hospital that provides free healthcare to the city (2018 population 800 064 [11]). Malawi is a low-income country in southeast Africa, with an estimated adult human immunodeficiency virus (HIV) prevalence of 9% [12] and a tuberculosis (TB) incidence of 133/100 000 person-years [13]. Blantyre has a subtropical climate with a rainy season from November to April. Malaria is endemic, peaking in the rainy season [14]. In this study, adults (aged ≥16 years) with sepsis were recruited from the emergency department from 7:00 am to 5:00 pm, Monday through Friday. As study design predated Sepsis-3 guidelines [1], inclusion criteria included evidence of infection (fever [axillary temperature >37.5°C] or history of fever within the preceding 72 hours) plus 1 or more of the following clinical markers of immediate organ dysfunction, locally predictive of poor outcome [15,16]: oxygen saturation <90%, respiratory rate >30 breaths/min, systolic blood pressure <90 mm Hg, or Glasgow coma score <15. These selection criteria were chosen because they are applicable in our setting and regionally generalizable. Individuals were excluded if they lacked the capacity to consent with no guardian available for proxy consent, spoke neither Chichewa nor English, and lived >30 km from Blantyre. Written informed consent was provided by the participant or, if they lacked capacity, their accompanying guardian. All care and treatment decisions for enrolled participants remained with the usual clinical team. Study team members followed participants hourly for the first 6 hours of hospital admission, then daily while an inpatient, and then in person at days 28, 90, and 180.

### Sampling and Laboratory Methods

Patients provided blood and urine at baseline and blood at day 28. Full details of tests/assays are provided in the Supplementary Methods. In brief, blood was tested for HIV-1/2 antibodies, *Plasmodium falciparum* histidine-rich protein-2 (HRP-2) antigen, standard biochemical and hematologic analyses, CD4 cell count quantification, automated aerobic culture, and mycobacterial culture (participants living with HIV/unknown HIV status only). Sputum testing for TB using Xpert Mycobacterium tuberculosis/rifampin (MTB/RIF) was carried out when there was a suspicion of pulmonary TB and cerebrospinal fluid (CSF) microscopy, culture, and lateral flow cryptococcal antigen testing when there was a suspicion of meningitis from the clinical team.

The following additional diagnostic tests were necessarily batched: urinary lipoarabinomannan (LAM; participants living with HIV/unknown HIV status only); testing of acute and convalescent sera for antibodies to chikungunya, dengue, and *Leptospira*; and convalescent sera for spotted fever group and epidemic typhus group rickettsioses. In addition, a subset of serum samples underwent polymerase chain reaction (PCR) for 46 bacterial and viral pathogens (Supplementary Methods). Case definitions are provided in Table 1. The study was designed and carried out before the WHO strong recommendation for LAM testing in seriously unwell inpatients living with HIV in high TB-burden settings [17]. HIV RNA testing was not available.

**Table 1.** Case Definitions Used in the Study

Diagnosis	Case Definition
Invasive bacterial infection	*Either*
	*Bloodstream infection* defined by culture of pathogenic bacteria from aerobic blood culture (with coagulase-negative Staphylococci, *Bacillus* spp., diphtheroids, and alpha-hemolytic Streptococci other than *Streptococcus pneumoniae* considered as contaminants) *OR* Detection of pathogenic bacterial DNA in blood by multiplex PCR
	*OR*
	*Meningitis* defined by culture of pathogenic bacteria from CSF (same definition of contaminants)
Invasive fungal infection	*Either*
	*Bloodstream infection:* Culture of fungus from blood
	*OR*
	*Meningitis:* Culture of fungus from CSF *OR* Detectible cryptococcal antigen by lateral flow assay in CSF
Tuberculosis^a^	*Either*
	*Mycobacterium tuberculosis* cultured from blood
	*OR*
	*M. tuberculosis* detected in sputum with Xpert MTB/RIF
	*OR*
	Detectible urinary lipoarabinomannan in urine by Alere lateral flow assay
Possible spotted fever group rickettsiosis	IgG convalescent serology titer ≥1:512
Possible epidemic typhus group rickettsiosis	
Chikungunya	Detectible IgM in either acute or convalescent serology or detectible pathogen DNA/RNA by PCR array
Dengue	
Leptospirosis	
All other diagnoses	Detectible pathogen DNA/RNA by PCR array (see Supplementary Methods for full list)

Abbreviations: CSF, cerebrospinal fluid; Ig, immunoglobulin; PCR, polymerase chain reaction.

^a^Pulmonary tuberculosis = positive Xpert MTB/RIF with no or negative urinary lipoarabinomannan (LAM) and/or mycobacterial blood culture). All other identified tuberculosis = disseminated (positive urinary LAM and/or positive mycobacterial blood culture irrespective of sputum Xpert MTB/RIF).

### Statistical Analyses

All analyses were carried out in R v4.0.2 (R Foundation for Statistical Computing, Vienna, Austria), and all models were fitted with Stan v2.21.0 via the R *brms* v2.13.5 package [18, 19].

The analysis aimed to identify modifiable treatment-related associations of sepsis mortality (full details are provided in the Supplementary Methods). In brief, we hypothesized a causal structure (Supplementary Figure 1) to identify which variables to control in order to estimate a causal effect of treatments on outcome. We used principal component analysis (PCA) to reduce the dimensionality of host-severity variables (Supplementary Table 1 and Supplementary Figure 8), and we used these along with untransformed infection and treatment variables as covariates in Bayesian logistic regression models with death by 28 days as the outcome. Missing data (which were infrequent; Supplementary Figure 10) were imputed with chained equations. Model outputs are presented as odds ratios (ORs) with a point estimate (posterior median) with 95% credible intervals (CrIs) or as marginal effect of the predictor on the probability of outcome with 95% CrIs for nonlinear models.

Finally, Kaplan-Meier plots were used to estimate the survival function over the study period, stratified by HIV status with hazard ratios (posterior median and 95% CrI) for living with HIV vs not living with HIV from a Bayesian Cox proportional hazards model to quantify the difference between the curves.

The University of Malawi College of Medicine and Liverpool School of Tropical Medicine research ethics committees approved the study, which is reported following the STrengthening the Reporting of OBservational studies in Epidemiology (STROBE) guidelines [20]. Data and code to replicate this analysis are available as the *BlantyreSepsis* R package at https://joelewis101.github.io/blantyreSepsis.

## RESULTS

### Baseline Characteristics

Between 19 February 2017 and 2 October 2018, 225 participants were recruited (Table 2, Supplementary Figure 2). There were 143 of 213 (67%) participants living with HIV. Antiretroviral therapy (ART) coverage was high. Of those living with HIV, 117 of 143 (82%) were on ART; 94% (110 of 117) were on the Malawian first-line regimen of efavirenz, lamivudine, and tenofovir disoproxil, for a median of 29 months (4–73). Despite this, CD4 counts were low for those on ART (median, 98; interquartile range [IQR], 31–236), and immunologic failure was common, with 52 of 84 (62%) participants on ART for longer than 6 months having a CD4 count <200 cells/mL. Participants living with HIV were older than participants not living with HIV; more likely to be female; and had been unwell for longer and with more severe disease at baseline as evidenced by a higher heart rate, lower blood pressure, and being less likely to be able to stand unaided (Supplementary Table 2).

**Table 2.** Characteristics of Included Participants

Variable	Value
Demographics	
Age, median (IQR), years	36 (28–44)
Male sex, n/N (%)	114/225 (51)
HIV/TB status	
Living with HIV,^a^ n/N (%)	143/213 (67)
CD4 lymphocyte count, median (IQR), 10^6^/L	156 (51–298)
Receiving antiretroviral therapy, n/N (%)	117/143 (82)
Time on antiretroviral therapy, median (IQR), months	29 (4–73)
Receiving co-trimoxazole preventative therapy, n/N (%)	98/141 (70)
History of receiving TB treatment, n/N (%)	37/225 (16)
Of those, currently receiving TB treatment, n/N (%)	10/37 (27)
Physiology	
Temperature, median (IQR), °C	38.5 (37.9–39.0)
Heart rate, median (IQR), beats/min	121 (102–132)
Respiratory rate, median (IQR), breaths/min	34 (32–38)
Systolic blood pressure, median (IQR), mm Hg	99 (85–119)
Diastolic blood pressure, median (IQR), mm Hg	66 (57–76)
Oxygen saturation, median (IQR), %	96 (94–98)
Glasgow coma score <15, n/N (%)	21/225 (9)
Unable to stand unaided, n/N (%)	63/225 (28)
Length of time unwell, median (IQR), days	7 (3–14)
Laboratory parameters	
Hemoglobin, median (IQR), g/dL	10.8 (8.2–13.2)
Platelets, median (IQR), 10^9^/L	218 (146–297)
White cell count, median (IQR), 10^9^/L	6 (4–11)
Sodium, median (IQR), mmol/L	134 (130–137)
Potassium, median (IQR), mmol/L	4.0 (3.6–4.4)
Bicarbonate, median (IQR), mmol/L	19 (17–22)
Creatinine, median (IQR), mmol/L	76 (59–103)
Lactate, median (IQR), mmol/L	3.4 (2.3–5.2)

Abbreviations: HIV, human immunodeficiency virus; IQR, interquartile range; TB, tuberculosis.

^a^HIV status missing for 12 participants.

### Treatments Received

The majority of participants (207 of 225, 92%) were administered broad-spectrum antibacterial agents (median, 5.3 hours [IQR, 3.7–10.8] from initial emergency department attendance), usually ceftriaxone (181 of 207, 87%; Supplementary Table 3) but also antituberculous (63 of 225, 28%), antifungal (26 of 225, 12%), or antimalarial (12 of 225, 5%) therapy. Median (IQR) duration of ceftriaxone was 5 days (2–7). Only patients with a positive malaria rapid test received antimalarial therapy. There were 192 of 225 (85%) participants who received intravenous fluid; a median of 1.5 L of fluid (IQR, 1.0–2.0 L) in the 6 hours following enrollment. None of the participants received inotropes, were intubated, or were admitted to the intensive care unit. Only 2 patients were switched to second-line ART during the 180-day study period.

### Etiology

A diagnosis was made 174 times in 144 of 225 (64%) participants (Table 3), most commonly TB in 34% (95% confidence interval [CI], 28%–41%). Acute rickettsioses and leptospirosis (2%; 95% CI, 1%–5% and 1%; 95% CI, 0–3% of participants) were uncommon. Evidence of past exposure to spotted fever group rickettsioses and chikungunya, however, was very common, with immunoglobulin G (IgG) detected in 61 of 147 (42%; 95% CI, 33%–50%) and 51 of 146 (35%; 95% CI, 27%– 43%) convalescent serum samples, respectively (Supplementary Table 4, Supplementary Figure 3).

**Table 3.** Diagnoses in Study Participants and Proportion of Participants With Positive Results

	Proportion of Participants With Positive Result			Cohort Prevalence		
Diagnosis	n/N	%	95% CI	n/N	%	95% CI
Bacterial infection (excluding mycobacteria)						
Bloodstream infection (culture)	24/224	11	(7%–15%)			
Bloodstream infection (polymerase chain reaction)	17/122	14	(8%–21%)			
Meningitis	0/44	0	(0%–8%)			
Any invasive bacterial infection	38/224	17	(12%–23%)	38/225	17	(12%–23%)
Possible rickettsioses						
Spotted fever group	2/147	1	(0%–5%)			
Epidemic typhus group	0/147	0	(0%–3%)			
Any rickettsiosis	2/147	1	(0%–5%)	2/225	1	(0%–3%)
Other						
Leptospirosis	2/179	1	(0%–4%)	2/225	1	(0%–3%)
Borreliosis	1/122	1	(0%–4%)	1/225	0	(0%–3%)
Mycobacterial infection						
Tuberculosis	76/162	47	(39%–55%)	76/225	34	(28%–41%)
Fungal infection						
Bloodstream infection	3/224	1	(0%–4%)			
Meningitis	4/44	9	(3%–22%)			
Any invasive fungal infection	5/224	2	(1%–5%)	5/225	2	(1%–5%)
Viral infection						
Arboviral infection						
Chikungunya	17/176	10	(6%–15%)			
Dengue	14/180	8	(4%–13%)			
Any arbovirus infection	31/182	17	(12%–24%)	31/225	14	(10%–19%)
Other						
Rift Valley fever	1/122	1	(0.05%)	1/225	0	(0%–3%)
Protozoal infection						
Falciparum malaria	21/219	10%	(6%–14%)	21/225	9	(6%–14%)

The denominator in the proportion of participants with positive results is the number of participants who received any diagnostic test for the given diagnosis. The denominator for cohort prevalence is all participants.

Abbreviation: CI, confidence interval.

The most common invasive bacterial pathogens identified on aerobic culture or PCR (43 pathogens in 38 participants; Supplementary Figures 4 and 5) were *Streptococcus* spp. (13 of 38 participants, 6 of which were pneumococcus), non-Salmonella Enterobacterales (12 of 38), and *Salmonella* spp. (11 of 38). Results of antimicrobial sensitivity testing are provided in Supplementary Figure 4. Considering only those participants living with HIV, 14 of 97 (14%) of those who reported taking cotrimoxazole preventative therapy (CPT) were diagnosed with an invasive bacterial infection compared with 7 of 43 (16%) of those not reporting taking CPT. All invasive fungal disease was caused by *Cryptococcus*, either *Cryptococcus neoformans* cultured from both blood and CSF (n = 3), CSF alone (n = 1), or detectible cryptococcal antigen in CSF (n = 1). Of the 76 diagnoses of TB, 2 were pulmonary (positive Xpert MTB/RIF only) and the rest disseminated; all but 1 of the participants with TB were living with HIV (Table 3). This patient group (disseminated TB) was clinically similar to the rest of the cohort (Supplementary Table 5). Of the 63 participants who received antituberculous therapy, 37 of 63 (59%) had a confirmed diagnosis of TB. Only 37 of 76 (49%) participants with TB received antituberculous therapy.

**Figure 1.**
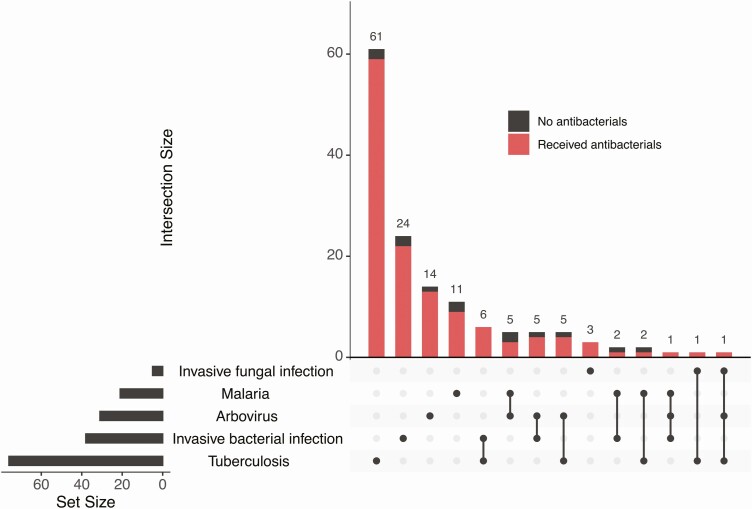
UpSet plot of diagnoses. Black circles in the lower half of the plot show diagnoses: either a single diagnosis (single circle) or 2 or more diagnoses (black circles linked by lines), with the large bar chart (top right) showing the number of participants with the indicated diagnosis or diagnoses. Only participants who had a diagnosis are included in this plot. The 5 most frequent diagnoses are shown, demonstrating that most participants had only 1 diagnosis. Red bar indicates receipt of antibacterial therapy, showing that almost all participants received antibacterial therapy despite no demonstrated invasive bacterial infection in many cases.

HIV infection predicted sepsis etiology (Table 4, Supplementary Table 6); malaria and arboviruses were more common in those not living with HIV (malaria: 17% vs 4%; difference, 13%; 95% CI, 4%–22%; arbovirus: 27% vs 6%; difference, 22%; 95% CI, 11%–33% for those living with and without HIV), whereas invasive fungal disease only occurred in participants living with HIV, and TB was more common in participants living with HIV (1% vs 50%; difference, 48%; 95% CI, 40%–57% for those living without and with HIV). Invasive bacterial infections occurred in similar proportions in participants living with and without HIV (20% vs 15%; difference, 5%; 95% CI, –5%–17%). While there were some coinfections, most participants with a diagnosis had a single etiologic agent (113 of 145, 78%: Figure 1, Supplementary Figure 6). Of the patients with a microbiologically confirmed diagnosis, only 35 of 145 (24%) were predicted to be susceptible to ceftriaxone, but 120 of 145 (83%) received it (Figure 1).

**Table 4.** Proportion of Participants With Diagnosis Stratified by Human Immunodeficiency Virus Status

Diagnosis	HIV+	HIV–	Difference (95% CI)
Arboviral infection	8/143 (6%)	19/70 (27%)	22% (11%–33%)
Invasive bacterial infection	21/143 (15%)	14/70 (20%)	5% (–5%–17%)
Invasive fungal infection	5/143 (3%)	0/70 (0%)	–3%( –7%––1%)
Malaria	6/143 (4%)	12/70 (17%)	13% (4%–22%)
Tuberculosis	71/143 (50%)	1/70 (1%)	–48% (–57%–40%)

Difference is proportion in participants not living with HIV minus proportion of participants living with HIV with bias-corrected bootstrap with 9999 replicates used to generate 95% CI.

Abbreviations: CI, confidence interval; HIV, human immunodeficiency virus.

### Outcome and Associations of Outcome

**Figure 2.**
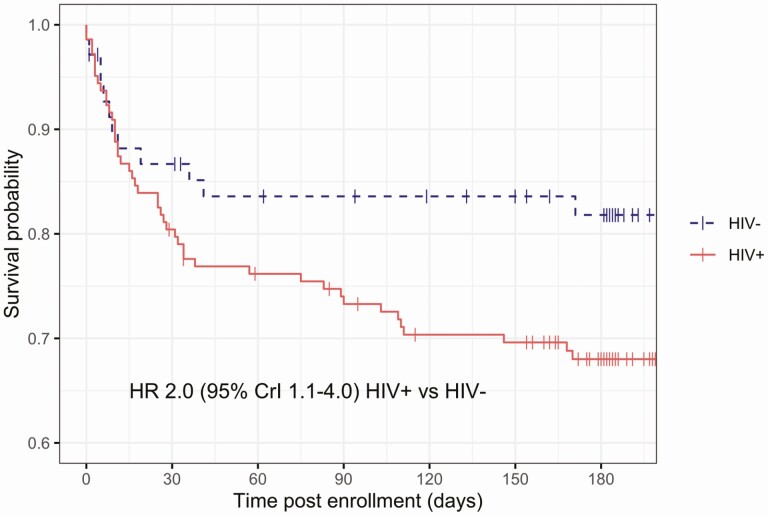
Kaplan-Meier estimate of survival function following sepsis admission, stratified by HIV status. Abbreviations: CrI, credible interval; HIV, human immunodeficiency virus; HR, hazard ratio.

Median (IQR) follow-up time was 182 days (62–202), a total of 92 person-years. The case fatality ratio (CFR) was 39 of 222 (18%; 95% CI, 13%–23%) at 28 days and continued to increase thereafter to 51 of 215 (24%; 95% CI, 18%–30%) at 90 days and 60 of 194 (31%; 95% CI, 25%–38%) at 180 days. In-hospital CFR was 14% (95% CI, 10%–19%) and median (IQR) length of hospitalization was 5 days (2–10). Early mortality was similar between participants living with and without HIV but diverged post-discharge (hazard ratio for death, 2.0; 95% CrI, 1.1–4.0 for participants living with and without HIV; Figure 2). Mortality was similar across diagnoses, though confidence intervals were wide (Table 5, Supplementary Figure 7). In an unadjusted analysis (Table 5), well-recognized host and sepsis severity variables and inability to stand were associated with death. Receipt of antimalarials and antituberculous chemotherapy were associated with survival in univariable analysis.

**Table 5.** Univariable Associations With Death by 28 Days

Variable	Died	Survived	Difference
Number of participants	39	183	-
Host variable			
Age, years	36.4 (31.5–46.0)	35.9 (27.4–42.9)	–0.5 (–8.1–3.2)
Male sex	19 (49%)	93 (51%)	2% (–16%–19%)
Living with human immunodeficiency virus^a^	27 (75%)	116 (67%)	–6% (–21%–11%)
**CD4 count, 10**^**6**^**/L**	**41 (17–225)**	**188 (69–302)**	**147 (46–186)**
**Hemoglobin,** ×**10**^**9**^**g/dL**	**9.1 (6.0–10.4)**	**11.0 (8.6–13.4)**	**1.9 (0.8–3.9)**
Severity variables			
**Temperature, °C**	**38.1 (37.7–38.8)**	**38.5 (38.0–39.0)**	**0.4 (0.0–0.7)**
Heart rate, beats/min	123 (105–139)	120 (102–131)	–3 (–13–6)
Systolic blood pressure, mm Hg	89 (76–121)	99 (87–119)	10 (–1–16)
**Diastolic blood pressure, mm Hg**	**60 (52–81)**	**67 (57–76)**	**7 (1–14)**
Respiratory rate, breaths/min	34 (32–37)	34 (32–38)	0 (–2–2)
**Oxygen saturation, %**	**95 (90–97)**	**97 (95–98)**	**2.0 (0–3)**
Glasgow coma score	15 (15–15)	15 (15–15)	0 (0–0)
**Unable to stand**	**27 (69%)**	**36 (20%)**	**–50% (–65%––33%)**
**Lactate, mmol/L**	**4.9 (3.0–10.6)**	**3.2 (2.1–4.5)**	**–1.7 (–6.2––0.1)**
White cell count, ×10^9^	5.9 (3.5–11.0)	6.9 (4.6–11.5)	1.0 (–1.4–2.8)
Platelet count, ×10^9^ /L	182 (87–301)	223 (148–297)	42 (–31–73)
Sodium, mmol/L	131 (127–138)	134 (130–137)	3 (–1–6)
**Bicarbonate, mmol/L**	**17** (14–21)	**20** (17–22)	**3 (0–5)**
**Urea, mmol/L**	**7.8 (4.5–14.3)**	**4.5 (3.2–7.0)**	**–3.3 (–8.7––1.3)**
Creatinine, mmol/L	90 (60–185)	73 (59–96)	–17 (–47–7)
Diagnosis			
Invasive bacterial infection	5 (13%)	32 (17%)	5% (–10%–15%)
Tuberculosis	15 (38%)	61 (33%)	–5% (–23%–10%)
**Malaria**	**0 (0%)**	**21 (11%)**	**11% (7%–16%)**
**Invasive fungal infection**	**3 (8%)**	**2 (1%)**	**–7% (–20%––1%)**
Chikungunya	2 (5%)	15 (8%)	3% (–9%–9%)
Dengue	2 (5%)	12 (6%)	1% (–10%–7%)
No diagnosis	18 (46%)	64 (35%)	–11% (–29%–6%)
Treatment received			
Antibacterials	37 (95%)	167 (91%)	–4% (–10%–7%)
Time to antibacterials, hours	4.7 (3.8–8.8)	5.3 (3.6–10.8)	0.6 (–1.1–1.7)
Antifungals	7 (18%)	19 (10%)	–8% (–23%–3%)
**Antimalarials**	**0 (0%)**	**12 (7%)**	**7% (3%–10%)**
**Antimycobacterials**	**6 (15%)**	**57 (31%)**	**16% (0%–27%)**
Intravenous fluid over 6 hours, L	1.4 (1.0–2.0)	1.3 (0.6–2.0)	–0.1 (–0.7–0.2)

Numeric variables are presented as median (interquartile range), and categorical variables are presented as proportions. Difference column shows difference in medians or difference in proportions with bias-corrected bootstrapped 95% confidence intervals (CIs). Variables shown in bold are those for which the 95% CIs do not cross 0.

^a^ Twelve participants had unknown human immunodeficiency virus status and are excluded from the denominator to calculate proportion; 3 died and 9 survived to 28 days.

**Figure 3.**
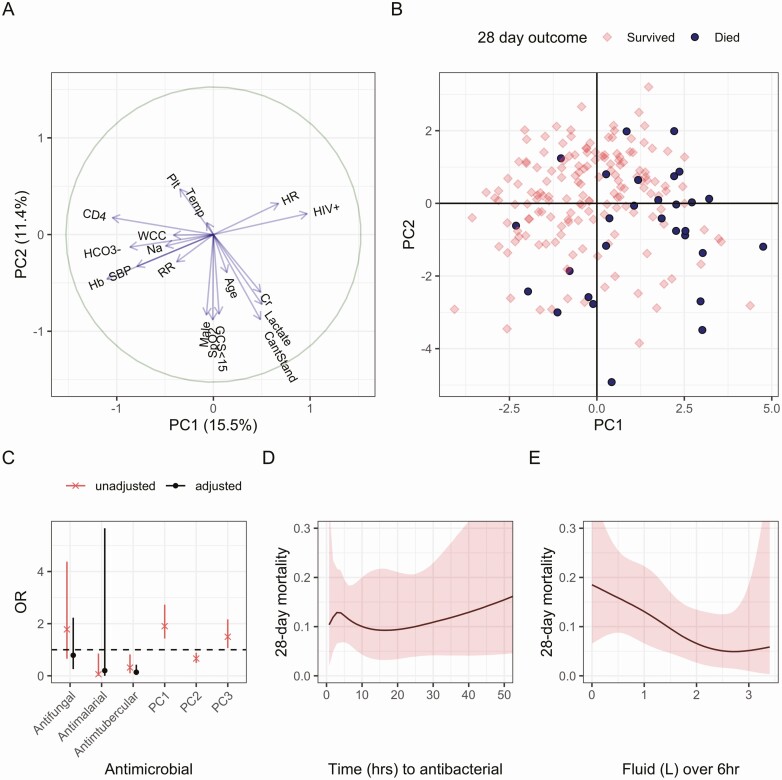
Determinants of sepsis mortality. *A,* Host severity PC1 and PC2 showing that PC1 defines an axis of HIV, immunosuppression (low CD4 count and anemia), and shock (tachycardia, low blood pressure, and bicarbonate), whereas PC2 is associated with sepsis-related organ dysfunction, age, and male sex. *B,* Participants projected onto PC1 and PC2 showing that participants who die (circles) tend to have immunosuppression and shock (upper right), other sepsis-related organ dysfunction (lower left), or both (lower right). *C–E*, Outputs of models predicting death by 28 days. Adjusted odds ratios and 95% credible intervals of effect of different antimicrobial therapies *(C)*, predicted mortality as a function of time to antibacterial therapy *(D)*, and volume of intravenous fluid received *(E)*. Abbreviations: Cr, creatinine; GCS, Glasgow coma score; Hb, hemoglobin; HIV, human immunodeficiency virus; HR, hazard ratio; OR, odds ratio; PC, principal component; RR, respiratory rate; SpO_2_, oxygen saturation; WCC, white cell count; Plt, platelet count.

We used Bayesian logistic regression with PCA-transformed host-severity variables (Supplementary Table 1) to estimate the effect of sepsis treatments received by study participants. Approximate leave-one-out cross-validation showed that when comparing models with 1 to 5 PCA coordinates as predictors of death, 3 coordinates had the best out-of-sample predictive value (quantified by expected log pointwise predictive density, ELPD; Supplementary Table 7). This model (using principal component [PC] 1, PC2, and PC3) was used as the base model to assess the effect of sepsis treatments on mortality. Together, PC1–PC3 explained 36% of the variance of the 18 included variables. PC1 defined an axis of HIV, immunosuppression, and shock; PC2 included severe sepsis organ dysfunction (low oxygen saturation, low Glasgow coma score, high lactate, and high creatinine), male sex, and inability to stand; and PC3 included thrombocytopenia (Figure 3A, Supplementary Figure 9). All 3 transformed variables, PC1–PC3, were associated with death by 28 days in the logistic regression models (Figure 3C, Supplementary Table 8).

Modeling the effect of different antimicrobial therapies showed a convincing association only between antituberculous therapy and survival (adjusted odds ratio [aOR] of death by 28 days, 0.17; 95% CrI, 0.05–0.49 for receipt of antituberculous therapy; Figure 3C, Supplementary Table 8). In linear models, the effect of 1 hour of antimicrobial delay on 28-day mortality was estimated to be aOR, 1.02 (95% CrI, 0.99–1.04), and the effect of 1 L of intravenous fluid to be aOR, 0.52 (95% CrI, 0.29–0.91; Supplementary Table 7). Relaxing the linearity assumption and allowing these relationships to be nonlinear with respect to time showed no convincing relationship between time to antimicrobial therapy and death (Figure 3D) but was suggestive of a lower mortality with increasing volume of administered fluid up to around 2 L (Figure 3E).

## DISCUSSION

We demonstrate that adults who present with sepsis in Blantyre, Malawi, are young compared with those in high-income settings, predominantly those living with HIV, and that their illness is caused by a heterogeneous group of pathogens, the majority of which are not susceptible to ceftriaxone. HIV status is the key determinant of sepsis etiology. Long-term outcomes are poor, driven by late mortality in those living with HIV. These data suggest several strategies that could improve sepsis outcomes in sub-Saharan Africa.

First, optimized antimicrobial strategies for sepsis in sub-Saharan Africa are needed; ceftriaxone would be expected to be effective in only 24% of participants with a diagnosis, yet 83% received this drug (median, 5 days). Ceftriaxone is a convenient, available drug in sub-Saharan Africa, but widespread use in Malawi [21] and regionally has been associated with an increase in antimicrobial resistance [22, 23]. Current diagnostic delays in establishing etiology necessitate immediate broad-spectrum antibacterial therapy. However, if novel sepsis protocols for sub-Saharan Africa can rapidly establish alternate diagnoses, perhaps with rapid diagnostic tests, rapid de-escalation of therapy and reduction of reliance on broad-spectrum antibacterials could be possible.

HIV status was the key determinant of sepsis etiology and represents a pragmatic starting point for management strategies. In particular, consideration of the need for antituberculous chemotherapy is indicated for people living with HIV who present with sepsis in high TB-burden settings. TB was the most common diagnosis in this study, in 34% of participants, and receipt of TB therapy was associated with improved survival (aOR of death, 0.17; 95% CrI, 0.05–0.49). A strategy of universal testing using lateral flow LAM testing of inpatients living with HIV was shown to improve survival in the STAMP trial [24], and the WHO recommends testing in all seriously ill inpatients living with HIV [17]. Even with expected improved sensitivity of the FujiLAM assay [25], however, LAM testing is likely to miss cases of disseminated TB. Furthermore, it can be challenging to get urine samples from critically ill patients in low-resource healthcare facilities, yet in the critically ill it may be that delaying antituberculous therapy is associated with poorer outcomes [8]. While empiric TB treatment has previously been assessed and has yet to demonstrate improved outcomes [26–28], none of these studies recruited inpatients with critical illness or sepsis. Our data suggest that there is a case for such interventional trials for critically unwell people living with HIV, perhaps based on presumptive TB therapy with step-down based on subsequent diagnostic testing.

In contrast, in people not living with HIV, malaria and arboviral infections are important causes of sepsis and more common than proven invasive bacterial infection. Indeed, the striking prevalence of chikungunya IgG seropositivity in study participants is comparable to a well-described outbreaks [29] and suggests widespread community transmission, which is in keeping with increased recognition of incidence of arboviral infections across sub-Saharan Africa. The rollout of rapid diagnostic tests for malaria has revolutionized the management of febrile illness in sub-Saharan Africa; these data suggest that there may be a role for rapid arboviral tests as well.

Second, post-discharge deaths must be addressed. We demonstrate significant long-term mortality following sepsis, driven by late deaths in participants living with HIV, perhaps due to ART failure and opportunistic infection. To our knowledge, these are the first estimates of case fatality ratios for sepsis in sub-Saharan Africa beyond 30 days. ART coverage in this cohort was high compared with historical cohorts [16, 30] and reflects the success of the Malawian ART program, thus a sepsis presentation is likely to be a manifestation of ART failure [31]. However, rates of switching to second-line therapy during the study period were very low. This may reflect programmatic challenges of documenting serial viral loads before switching, per WHO guidance [32]. Structural interventions that optimize the identification of ART failure in acute illness and rapidly switch to second-line therapy should be investigated.

There are limitations to our study, especially around our ability to deliver comprehensive or real-time diagnoses. It was only possible to carry out urinary LAM testing at the end of the study [17]. Because serologic testing was largely carried out on convalescent sera, we could not relate serologic diagnoses to survival. Testing IgG on paired sera would likely most accurately classify diagnoses but was not possible because of resource limitations; serologic diagnoses of arboviruses and leptospirosis were based on IgM, which may be nonspecific. Rickettsial IgM, in particular, has poor specificity, so an IgG-based case definition was used, which may misclassify acute cases. Funding did not permit identification of some causes of febrile illness, for example, *Coxiella* serology for Q fever or *Pneumocystis jirovecii*. We could not establish late causes of mortality or provide HIV viral load testing. For a malaria diagnosis, we used HRP-2 antigenemia rather than smear, which could reflect recent but not current malaria. In addition, a number of streptococcal diagnoses were made using PCR, which may have poor specificity [33]. Though we have used a principled modeling approach with an explicit hypothesized causal structure to identify associations of mortality, it is likely that residual confounding remains. The dataset is too small to model all treatments and diagnoses simultaneously or to explore interaction effects (eg, explore whether treatments are beneficial only in groups with confirmed disease). Though Blantyre is likely to be reasonably representative of an urbanizing population in sub-Saharan Africa, the study was carried out at a single center.

In conclusion, sepsis in Blantyre, Malawi, is caused by a diverse group of pathogens including *M. tuberculosis*, arboviruses, and malaria, yet management relies on prolonged courses of broad-spectrum antibacterial agents that would not treat these pathogens. HIV status is a key determinant of outcome and can assist clinicians in targeting antimicrobial therapy. Long-term outcomes are poor and driven by late deaths in people living with HIV. Understanding the reasons for these late deaths is of prime importance. Optimization and operationalization of treatment solutions in sub-Saharan Africa will require both randomized, controlled trials and pragmatic implementation science approaches, but the high mortality in these patient populations represents an important opportunity to improve outcomes.

## Supplementary Data

Supplementary materials are available at *Clinical Infectious Diseases* online. Consisting of data provided by the authors to benefit the reader, the posted materials are not copyedited and are the sole responsibility of the authors, so questions or comments should be addressed to the corresponding author.

ciab710_suppl_Supplementary_Tables_and_Figures

ciab710_suppl_Supplementary_Methods

ciab710_suppl_Supplementary_Material

## Notes

***Acknowledgments.*** The authors thank the study team: Tusekile Phiri, Grace Mwaminawa, Witness Mtambo, Gladys Namacha, and Monica Matola; the Malawi-Liverpool Wellcome (MLW) laboratory teams, particularly Brigitte Denis; and the MLW data team, particularly Lumbani Makhaza and Clemens Masesa. They also thank those at the Rare and Imported Pathogens Laboratory: Jennifer Small for Immunofluorescence work and Kate Withycombe and Supriya Gurung for the enzyme-linked immunosorbent assay work.

***Financial support.*** This work was supported by the Wellcome Trust (109105z/15/a to J. M. L. and 206545/Z/17/Z, the core grant to the Malawi-Liverpool-Wellcome Programme).

***Potential conflicts of interest.*** J. R. reports an National Institute for Health Research (NIHR) Global Health Research Group on Sepsis at the Liverpool School of Tropical Medicine, African Research Collaboration on Sepsis (ARCS) grant through their institution Liverpool School of Tropical Medicine (LSTM) and a Clinical Research Career Development Fellowship grant through their institution (LSTM). All other authors report no potential conflicts. All authors have submitted the ICMJE Form for Disclosure of Potential Conflicts of Interest. Conflicts that the editors consider relevant to the content of the manuscript have been disclosed.
