# Supplementary material for: A Longitudinal, Observational Study of Etiology and Long-Term Outcomes of Sepsis in Malawi Revealing the Key Role of Disseminated Tuberculosis
Source: Clin Infect Dis. 2021 Aug 18;74(10):1840–9. doi: 10.1093/cid/ciab710 (PMC9155594; doi:10.1093/cid/ciab710)
Supplement: ciab710_suppl_Supplementary_Tables_and_Figures [file ciab710_suppl_supplementary_tables_and_figures.pdf]

A longitudinal observational study of aetiology and long-term outcomes of sepsis in Malawi revealing the key role of disseminated tuberculosis

SUPPLEMENTARY RESULTS

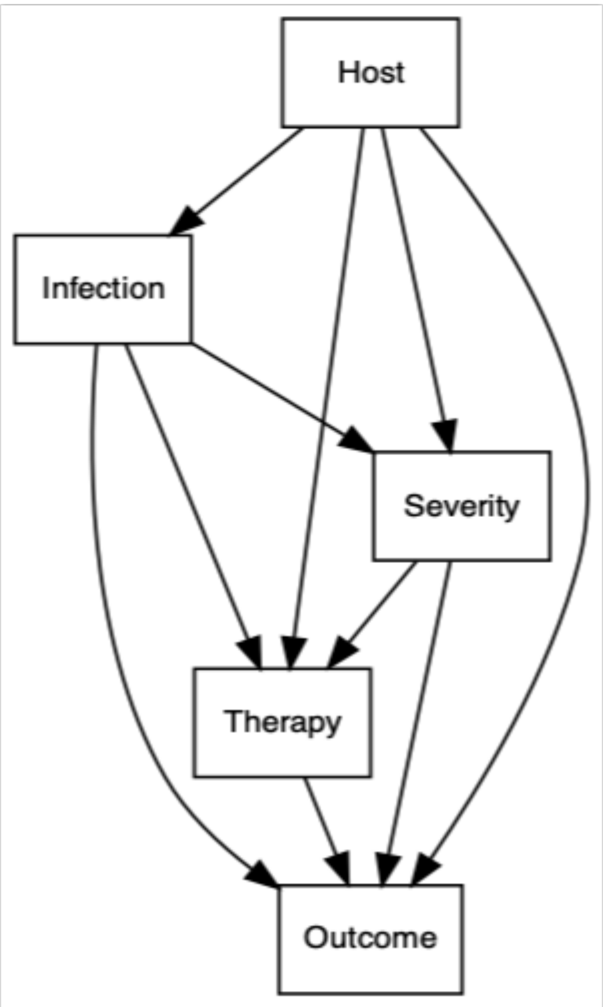

Supplementary Figure E1: Hypothesized causal structure for outcome in sepsis. Host variables would include HIV status, measures of immunosuppression, sex and age. Infection variables would encode the pathogen(s) causing sepsis. Severity variables would include measures of sepsis severity (e.g. blood pressure, lactate). Therapy variables would include antimicrobials administered, intravenous fluids. Outcome variables could be mortality (e.g. death by 28 days) or morbidity.

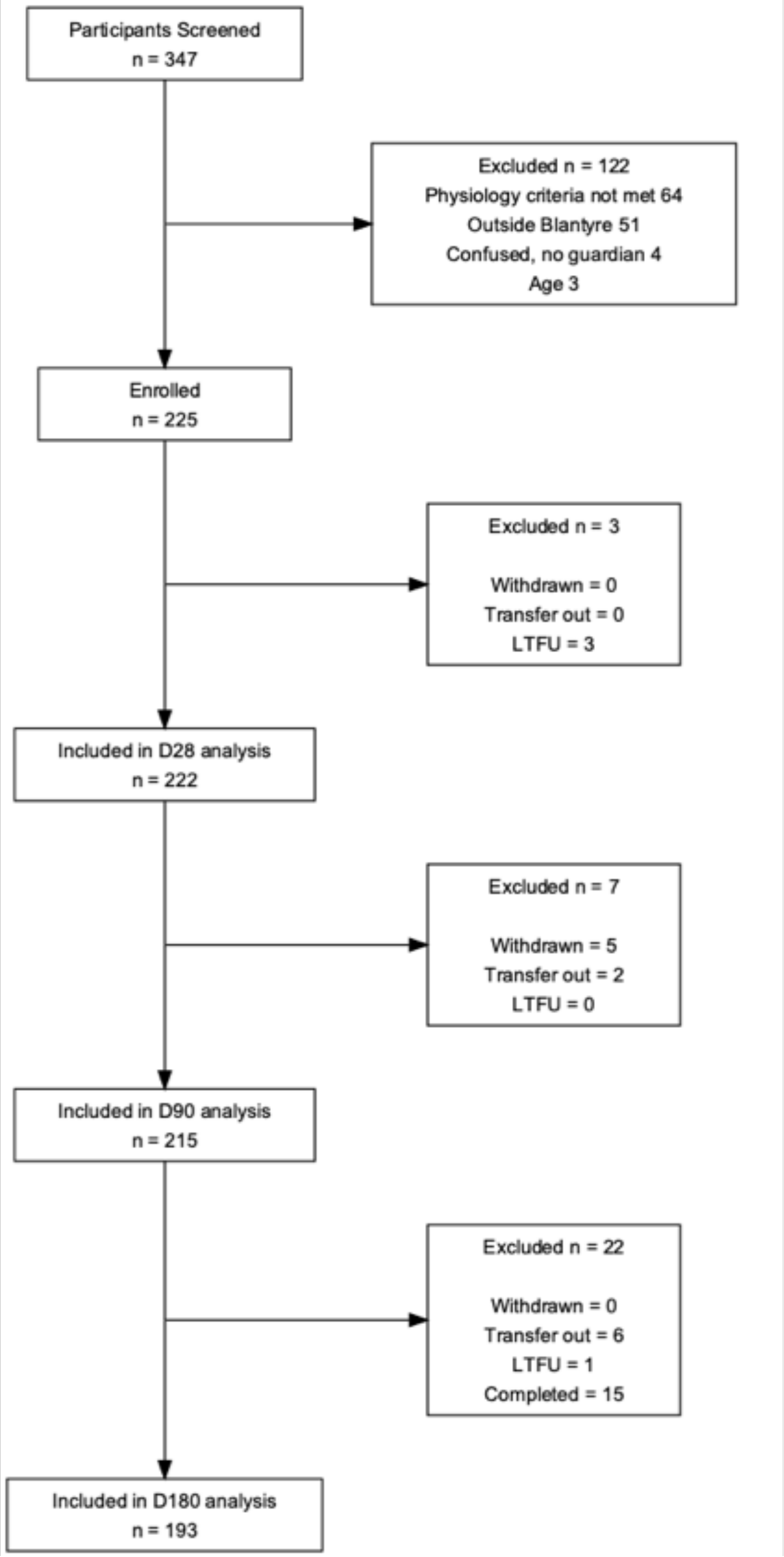

Supplementary Figure E2: Flow through the study. 15 participants completed their final visit prior to 180 days and are not included in the calculation of 180 day mortality.

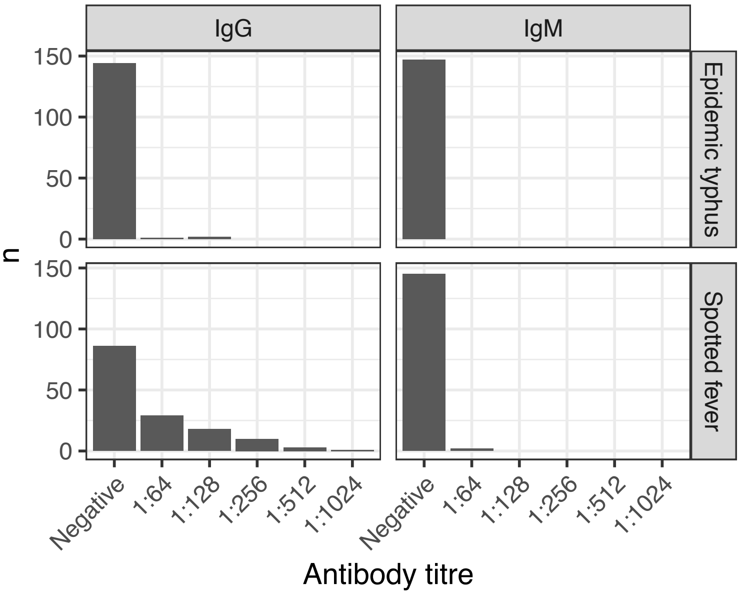

Supplementary Figure E3: Day 28 Anti-Rickettsia antibody titres to Epidemic typhus (top) and Spotted fever group Rickettsioses

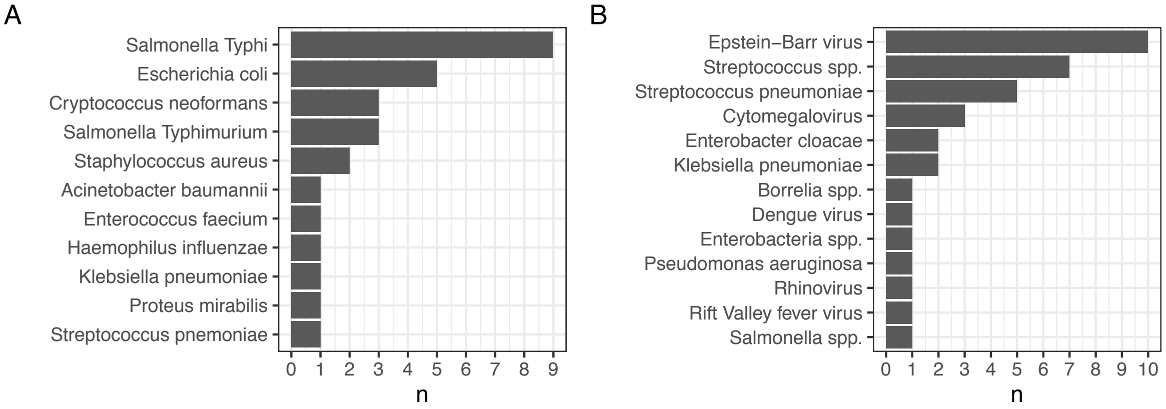

Supplementary Figure E4: Pathogens identified by (A) aerobic blood culture and (B) multiplex PCR on blood. 26 blood cultures in 24/224 participants grew 28 pathogens in total. 26 pathogen PCR targets were identified in 31/122 samples. n here is number of samples. Antimicrobial susceptibility testing was available for all isolates grown in aerobic blood culture: all *Salmonella* spp. (n=10) and *S. pneumoniae* (n=1) were susceptible to ceftriaxone, but 2/7 (29%) non-Salmonella Enterobacterales, were extended-spectrum beta-lactamase (ESBL) producers, and resistant, both from the same participant. In total, of the 38 participants with proven invasive bacterial infection, 5 (13%) would be expected to be ceftriaxone non-susceptible either due to intrinsic resistance (n=2), chromosomal AmpC (n=2) or ESBL production (n=1).

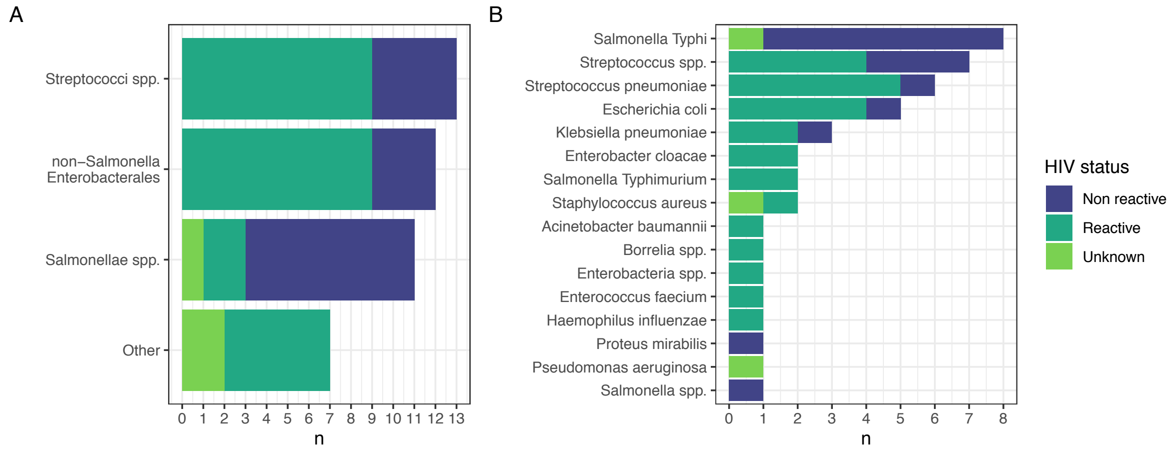

Supplementary Figure E5: number of participants in whom a bacterial pathogen was identified: aggregated (A) and to species level (B). 43 bacterial pathogens were identified in 38 participants. n here is number of participants.

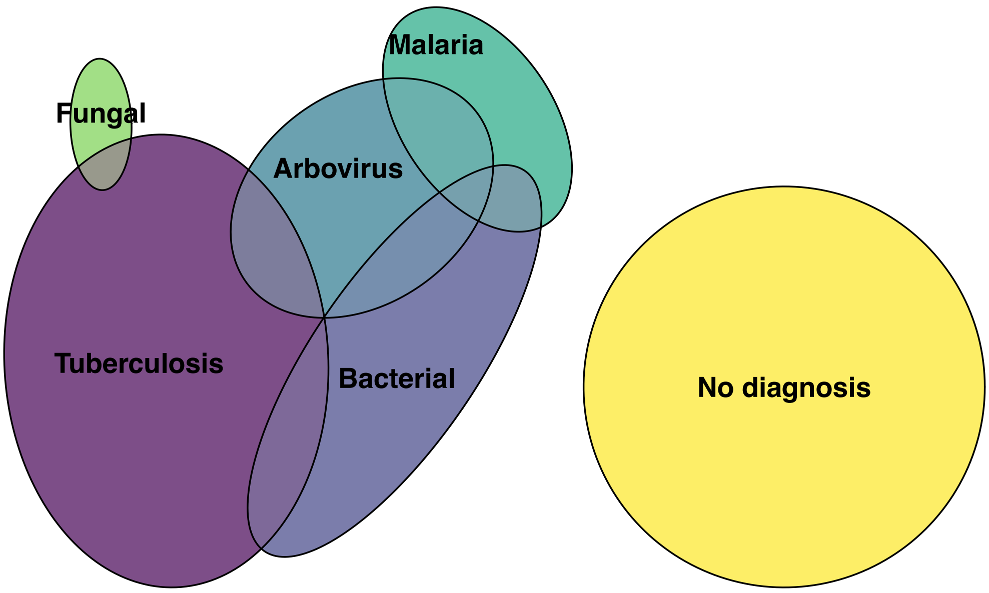

Supplementary Figure E6: Euler diagram showing overlapping diagnoses.

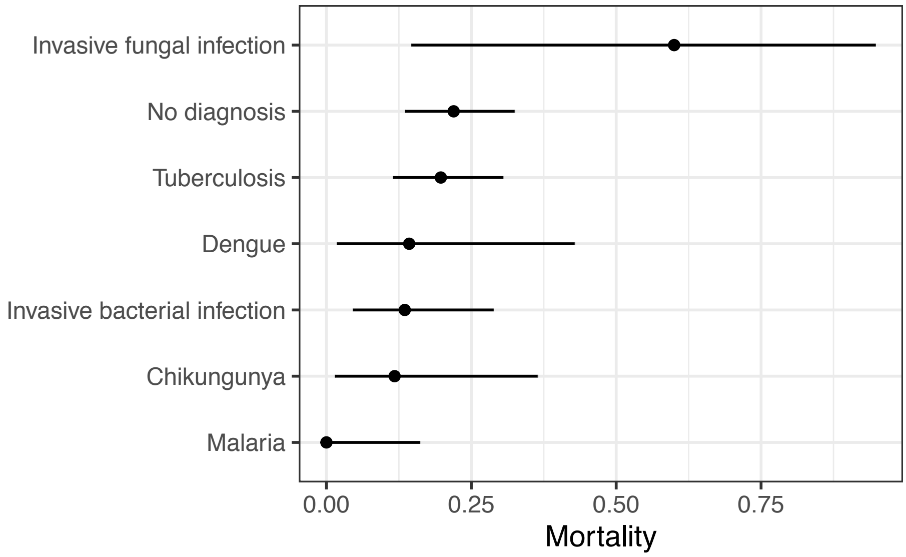

Supplementary Figure E7: Mortality stratified by diagnosis

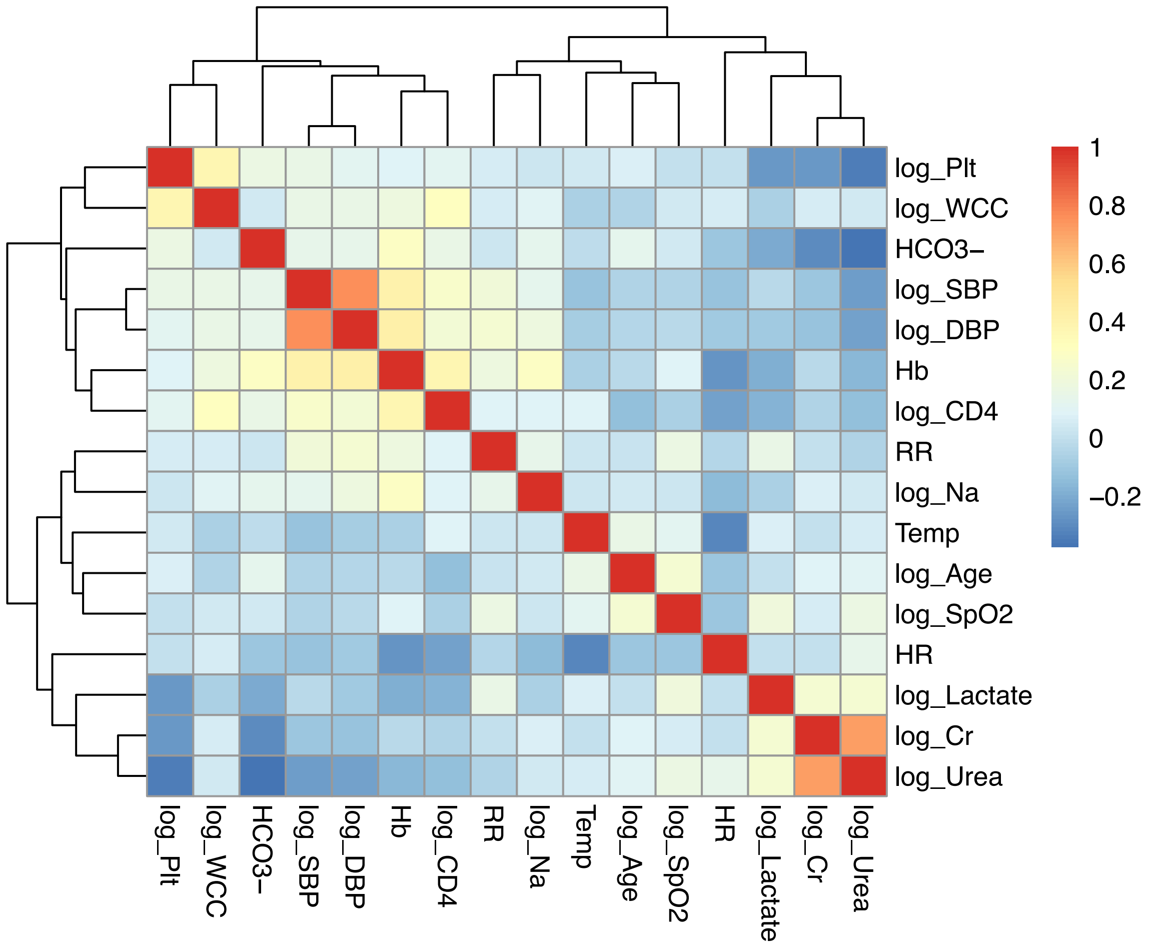

Supplementary Figure E8: Correlation matrix of included continuous variables (using Pearson's correlation coefficient), row- and column-clustered using hierarchical clustering, showing very strong correlation between creatinine and urea and systolic and diastolic blood pressure. Also note negative correlation (though less strong) between renal failure and thrombocytopenia and low bicarbonate. Plt = Platelet count, WCC= White cell count, HCO3- = serum bicarbonate, SBP = systolic blood pressure, DBP = diastolic blood pressure, Hb = Haemoglobin, CD4 = CD4 cell count, RR = respiratory rate, Na = Serum sodium, Temp = Temperature, SpO2 = Capillary oxygen saturation, HR = Heart rate, Cr = Creatinine. Prefix log_ indicates log transformation of variable.

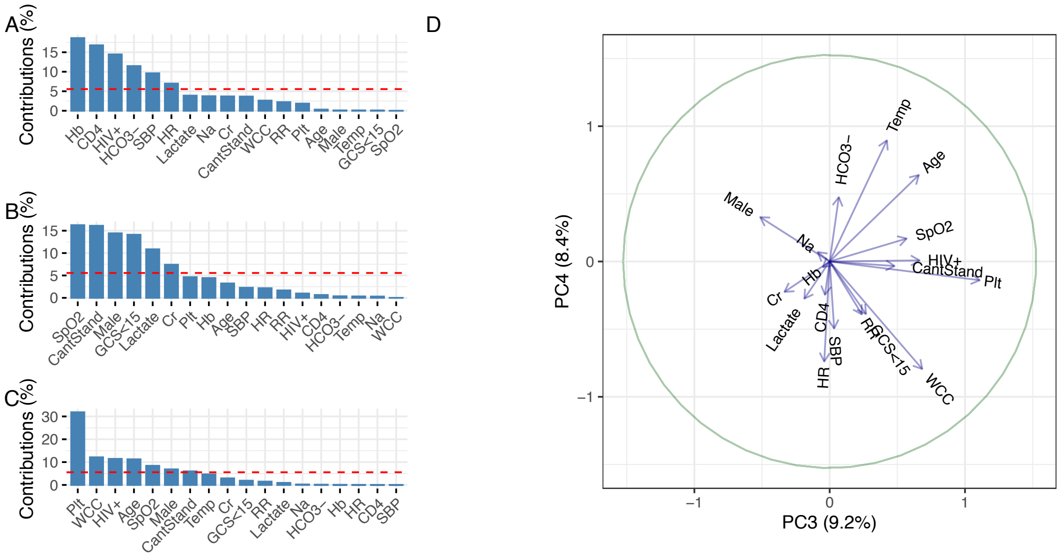

Supplementary Figure E9: Original host-severity variable contributions to PC1 (A), PC2 (B) and PC3 (C) and original variables projected onto PC3 and PC4 (D).

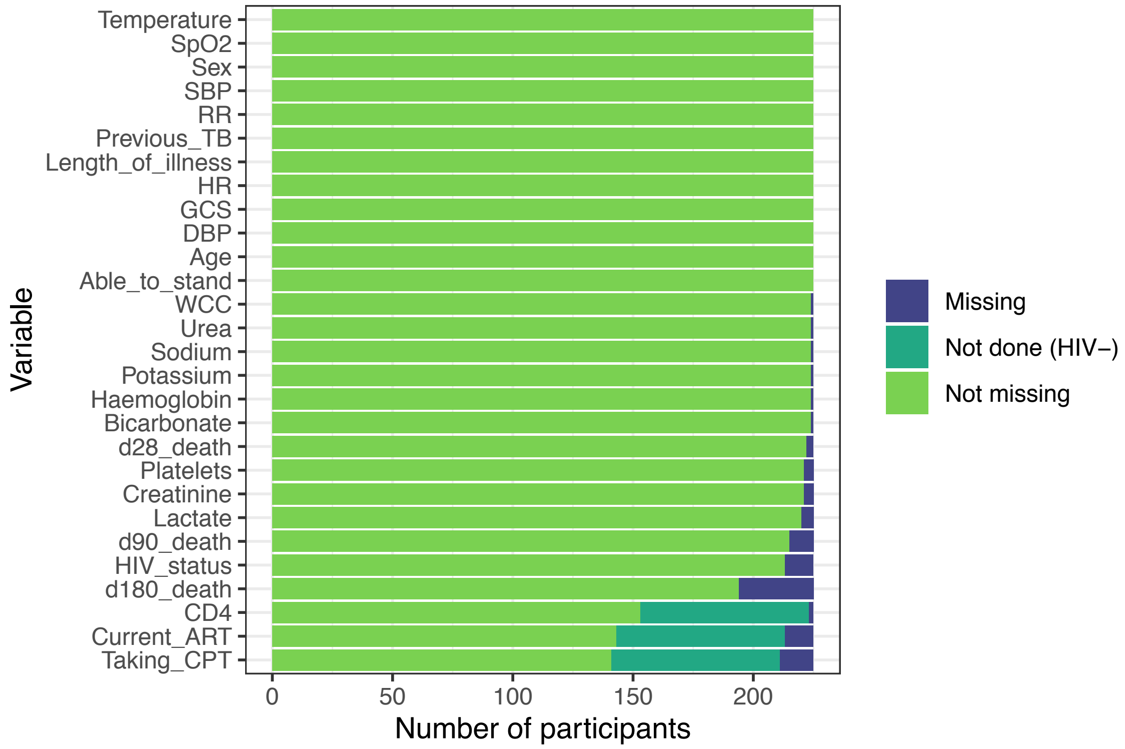

Supplementary Figure E10: Missing data by variable. HIV related variables were not available for HIV-noninfected participants, recorded here as “Not done (HIV-)”. SpO2 = capillary oxygen saturation, SBP/DBP = systolic/diastolic blood pressure, RR = respiratory rate, TB = tuberculosis, WCC = white cell count, ART = antiretroviral therapy, CPT = cotrimoxazole preventative therapy.

Supplementary Table E1: Included host-severity variables, definitions, and transformations.

Variable name	Description	Transformation
CantStand	Binary, {1 = unable to stand, 0 = able to stand}	None
Hb	Continuous, Blood haemoglobin in g/dL	None
HR	Continuous, heart rate in beats/min	None
RR	Continuous, respiratory rate in beats/min	None
HCO3-	Continuous, serum bicarbonate in mmol/L	None
Male	Binary, {1 = male, 0 = female}	None
HIV+	Binary, {1 = HIV infected, 0 = not HIV infected}	None
GCS<15	Binary, {1 = GCS < 15, 0 = GCS = 15}	None
Age	Continuous, age in years at enrolment	Log (x)
CD4	Continuous, CD4 cell count in cells/mL	Log (x)
Cr	Continuous, serum creatinine in mmol/L	Log(x)
Lactate	Continuous, serum lactate in mmol/L	Log (x)
Plt	Continuous, blood platelet count in $x10^9$ cells/ L	Log (x)
SpO2	Continuous, capillary oxygen saturation, %	Log (101-x)
SBP	Continuous, systolic blood pressure in mmHg	Log (x)
Temp	Continuous, temperature in	log(41 -x)
WCC	Continuous, blood white cell count in in $x10^9$ cells/ L	Log (x)
Na	Continuous, serum sodium in mmol/L	Log (x)

All log transformations use natural log, base *e*. GCS = Glasgow coma score.

Supplementary Table E2: Univariable baseline associations with HIV status.

**Variable**	**HIV infected**	**HIV uninfected**	**Difference**
Number of participants	143	70	-
**Age (years)**	**38.0 (32.3-43.9)**	**32.0 (24.0-39.6)**	**-6.0 (-11.6 to -2.9)**
**Male sex**	**62 (43%)**	**44 (63%)**	**20% (5 to 33%)**
**Length of time unwell for (days)**	**10.0 (4.0-21.0)**	**6.5 (3.0-14.0)**	**-3.5 (-10.0 to 0.0)**
**Previous or current TB**	**35 (24%)**	**2 (3%)**	**-22% (-30 to -14%)**
Temperature (C)	38.4 (37.9-39.0)	38.5 (38.0-39.2)	0.1 (-0.1 to 0.4)
**Heart rate (beats/min)**	**124.0 (110.0-134.0)**	**111.5 (98.0-129.2)**	**-12.5 (-22.0 to -6.0)**
**Systolic BP (mmHg)**	**95.0 (83.5-113.0)**	**104.0 (92.8-123.0)**	**9.0 (2.0 to 25.5)**
**Diastolic BP (mmHg)**	**65.0 (55.0-72.0)**	**72.0 (62.2-84.8)**	**7.0 (1.0 to 11.0)**
Respiratory rate (breaths/min)	34.0 (32.0-38.0)	34.0 (32.0-36.0)	0.0 (-2.0 to 2.0)
Oxygen saturation (%)	97.0 (94.0-98.0)	96.0 (95.0-98.0)	-1.0 (-1.0 to 1.0)
GCS below 15	13 (9%)	7 (10%)	1% (-7 to 10%)
**Unable to stand**	**44 (31%)**	**12 (17%)**	**-14% (-25 to -2%)**
**Haemoglobin ($x10^9$} g/dL)**	**10.0 (7.6-11.6)**	**13.3 (10.9-15.0)**	**3.3 (2.1 to 4.2)**
White cell count ($x10^9$)	6.4 (4.2-11.8)	6.7 (4.6-11.0)	0.2 (-1.3 to 2.0)
Platelet count ($x10^9$ /L)	230.0 (156.0-314.0)	202.0 (125.8-288.0)	-28.0 (-78.5 to 6.0)
**Sodium (mmol /L)**	**133.0 (128.0-136.0)**	**135.0 (133.0-138.0)**	**2.0 (0.5 to 4.0)**
**Bicarbonate (mmol /L)**	**19.0 (16.0-21.0)**	**21.5 (18.2-24.0)**	**2.5 (1.0 to 4.0)**
Creatinine (mmol /L)	75.0 (58.0-105.0)	73.5 (60.2-95.0)	-1.5 (-9.0 to 12.0)
Lactate (mmol/L)	3.7 (2.4-5.2)	3.1 (2.1-5.0)	-0.6 (-1.0 to 0.2)

Numeric variables are presented as median (IQR) and categorical variables as proportions. Difference column shows difference in medians or difference in proportions with 95% confidence intervals. Variables shown in bold are those for which the 95% confidence intervals do not cross 0.

Supplementary Table E3: Antimicrobial therapies received by study participants.

Antimicrobial therapy	No. (proportion [95% CI]) participants	Median (IQR) hours to administration
**Antibacterial**	**207/225 (92% [88-95%])**	**5.3 (3.7-10.8)**
Ceftriaxone	181/207 (87%)	
Ciprofloxacin	18/207 (9%)	
Amoxicillin	5/207 (2%)	
Metronidazole	2/207 (1%)	
Co-amoxiclav	1/207 (0%)	
**Antitubercular**	**63/225 (28% [22-34%])***	**120.9 (63.7-171.0)**
RHZE	63/63 (100%)	
**Antifungal**	**26/225 (12% [8-16%])**	**47.7 (27.9-73.9)**
Fluconazole	25/26 (96%)	
Amphotericin	1/26 (4%)	
**Antimalarial**	**12/225 (5% [3-9%])**	**4.5 (3.1-21.7)**
Artesunate	11/12 (92%)	
Lumefantrine-Artemether	1/12 (8%)	

* 10/63 participants who received antitubercular agents during admission were taking them prior to admission; they are excluded from the calculation of median door-to-antimicrobial time for this class. RHZE = standard antituberculous chemotherapy: Rifampicin (R), Isoniazid (H), Pyrazinamide (Z) and Ethambutol (E)

Supplementary Table E4: Proportion of participants with detectible IgG and IgM to the selected pathogens in day 0 and day 28 serum.

	Day 0		Day 28	
**Organism**	**IgG**	**IgM**	**IgG**	**IgM**
Chikungunya	16/41 (39.0% [24.2-55.5])	9/41 (22.0% [10.6-37.6])	51/146 (34.9% [27.2-43.3])	15/143 (10.5% [6.0-16.7])
Dengue	1/36 (2.8% [0.1-14.5])	5/36 (13.9% [4.7-29.5])	12/144 (8.3% [4.4-14.1])	13/147 (8.8% [4.8-14.6])
Leptospira	–	0/35 (0.0% [0.0-10.0])	–	2/146 (1.4% [0.2-4.9])
Epidemic typhus group	–	–	3/147 (2.0% [0.4-5.8])	0/147 (0.0% [0.0-2.5])
Spotted fever group	–	–	61/147 (41.5% [33.4-49.9])	2/147 (1.4% [0.2-4.8])

NB: Denominator in each case is number of participants who had a given test carried out. There were 8 acute-convalescent sera pairs which were tested for Chikungunya, and 3 which were tested for Dengue. There were no IgG seroconversions identified.

Supplementary Table E5: Univariable associations with diagnosis of tuberculosis.

Variable	TB	no TB	Difference
Age (years)	37.2 (31.1-42.7)	35.3 (26.3-44.2)	-1.9 (-5.0 to 2.6)
Male sex	37 (49%)	77 (52%)	3% (-11 to 17%)
**Length of time unwell for (days)**	**14.0 (6.0-28.5)**	**7.0 (3.0-14.0)**	**-7.0 (-9.0 to 0.0)**
**HIV Infected**	**71 (99%)**	**72 (51%)**	**-45% (-54 to -35%)**
**CD4 count 10^6 /L**	**100.0 (35.5-209.5)**	**215.0 (67.5-334.0)**	**115.0 (57.0 to 158.0)**
Temperature (C)	38.5 (38.0-39.0)	38.5 (37.9-39.0)	0.0 (-0.2 to 0.3)
**Heart rate (beats/min)**	**125.0 (117.8-136.2)**	**116.0 (98.0-131.0)**	**-9.0 (-17.0 to -4.0)**
Systolic BP (mmHg)	93.0 (85.0-111.5)	99.0 (87.0-120.0)	6.0 (-4.0 to 12.0)
Diastolic BP (mmHg)	67.0 (52.8-72.0)	66.0 (57.0-77.0)	-1.0 (-4.5 to 7.0)
Respiratory rate (breaths/min)	34.0 (32.0-38.0)	34.0 (32.0-36.0)	0.0 (-2.0 to 2.0)
Oxygen saturation (%)	97.0 (94.0-98.0)	96.0 (95.0-98.0)	-1.0 (-1.0 to 0.5)
GCS	15.0 (15.0-15.0)	15.0 (15.0-15.0)	0.0 (0.0 to 0.0)
Unable to stand	23 (30%)	40 (27%)	-3% (-17 to 9%)
**Haemoglobin ($x10^9$} g/dL)**	**8.9 (6.6-11.0)**	**11.5 (9.5-13.9)**	**2.6 (1.3 to 3.7)**
White cell count ($x10^9$)	6.3 (4.2-8.9)	6.8 (4.5-11.7)	0.5 (-0.8 to 2.3)
Platelet count ($x10^9$ /L)	216.0 (146.5-299.0)	218.5 (145.8-295.5)	2.5 (-50.5 to 50.0)
**Sodium (mmol /L)**	**132.0 (127.0-135.5)**	**134.0 (132.0-137.0)**	**2.0 (1.0 to 6.0)**
**Bicarbonate (mmol /L)**	**18.0 (14.5-20.0)**	**20.0 (17.0-23.0)**	**2.0 (0.0 to 3.0)**
Urea (mmol /L)	4.8 (3.5-8.2)	4.7 (3.4-7.7)	-0.1 (-1.7 to 0.8)
Creatinine (mmol /L)	74.5 (55.8-100.5)	78.0 (60.0-103.0)	3.5 (-5.5 to 17.0)
Lactate (mmol/L)	3.2 (2.3-5.2)	3.4 (2.2-5.1)	0.2 (-0.7 to 0.8)

Numeric variables are presented as median (IQR) and categorical variables as proportions. Difference column shows difference in medians or difference in proportions with 95% confidence intervals. Variables shown in bold are those for which the 95% confidence intervals do not cross 0.

Supplementary Table E6: Positive diagnostic test results stratified by HIV status.

	HIV Status		
**Test**	**Infected**	**Non-infected**	**Unknown**
Number of participants	70	143	12
**TB diagnostics***			
Urine LAM-LF	–	70/136 (51% [43-60])	4/9 (44% [14-79])
Sputum Xpert MTB/RIF	1/8 (12% [0-53])	7/35 (20% [8-37])	0/1 (0% [0-98])
Blood culture	–	7/128 (5% [2-11])	1/10 (10% [0-45])
**Bacterial diagnostics**			
Aerobic blood culture	14/70 (20% [11-31])	21/142 (15% [9-22])	3/12 (25% [5-57])
CSF culture	0/12 (0% [0-26])	0/31 (0% [0-11])	0/1 (0% [0-98])
PCR: Bacterial pathogen DNA detected	5/49 (10% [3-22])	11/66 (17% [9-28])	1/7 (14% [0-58])
**Arboviral diagnostics**			
Chikungunya IgM	8/47 (17% [8-31])	5/120 (4% [1-9])	3/9 (33% [7-70])
Dengue IgM	10/50 (20% [10-34])	3/121 (2% [1-7])	1/9 (11% [0-48])
**Malaria diagnostics**			
P. falciparum RDT	12/69 (17% [9-28])	6/138 (4% [2-9])	3/12 (25% [5-57])
**Fungal diagnostics**			
Aerobic blood culture	0/70 (0% [0-5])	3/142 (2% [0-6])	0/12 (0% [0-26])
CSF culture	0/12 (0% [0-26])	4/31 (13% [4-30])	0/1 (0% [0-98])
CSF CrAg	0/6 (0% [0-46])	4/23 (17% [5-39])	–
**Rickettsial diagnostics**			
Spotted fever IgG ≥ 1:512	2/45 (4% [1-15])	2/94 (2% [0-7])	0/8 (0% [0-37])
Epidemic typhus IgG ≥ 1:512	0/45 (0% [0-8])	0/94 (0% [0-4])	0/8 (0% [0-37])
**Leptospirosis diagnostics**			
Leptospirosis IgM	1/50 (2% [0-11])	1/120 (1% [0-5])	0/9 (0% [0-34])
**PCR Array card**			
PCR: Borrelia DNA detected	0/49 (0% [0-7])	1/66 (2% [0-8])	0/7 (0% [0-41])
PCR: Rift valley fever virus detected	1/49 (2% [0-11])	0/66 (0% [0-5])	0/7 (0% [0-41])

* Urinary LAM testing and mycobacterial blood culture testing were only carried out in HIV infected or HIV unknown participants. TB = Tuberculosis, LAM-LF =

Lipoaribomannan lateral flow, CSF = Cerebrospinal fluid, RDT = rapid diagnostic test, CrAg = Cryptococcal antigen.

Supplementary Table E7: Model out-of-sample predictive accuracy quantified by the expected log pointwise predicted density, and quantified by leave-one-out cross validation $ELPD_{loo}$.

	$ELPD_{loo}$	$ELPD_{loo}$ difference	Standard error of $ELPD_{loo}$ difference
Model 3 (PC1-3)	-69.1	0	0
Model 4 (PC1-2)	-70.0	-0.9	3.2
Model 2 (PC1-4)	-70.1	-1.0	0.2
Model 1 (PC1-5)	-70.7	-1.6	1.7

Models use between 2 and 5 of the principal component transformed variables, with the difference in $ELPD_{loo}$ to model 3 shown, as well as the standard error of this difference. A smaller (closer to 0) value of $ELPD_{loo}$ is better; therefore model 3 has the best predicted predictive accuracy. In all cases, however, the standard error of the difference is of a similar magnitude as the difference; we conclude that we cannot be confident that any one model is better than the others.

Supplementary Table E8: Parameter estimates from models assessing effect of therapies on mortality, expressed as adjusted odds ratios with a point estimate (posterior median) and 95% credible intervals.

		Model				
**Parameter**	**Unadjusted**	**TB treatment**	**Malaria treatment**	**Fungal treatment**	**Time to anti-bacterial**	**Vol of IV fluid**
PC1	1.90 (1.43-2.73)	2.26 (1.69-3.18)	1.92 (1.47-2.61)	1.98 (1.50-2.74)	1.97 (1.51-2.71)	2.36 (1.73-3.42)
PC2	0.66 (0.49-0.88)	0.60 (0.44-0.79)	0.61 (0.46-0.80)	0.62 (0.47-0.80)	0.64 (0.48-0.83)	0.59 (0.44-0.77)
PC3	1.50 (1.06-2.17)	1.32 (0.98-1.83)	1.21 (0.88-1.68)	1.27 (0.93-1.75)	1.31 (0.97-1.81)	1.36 (1.10-1.88)
Diagnosis is TB	1.22 (0.59-2.48)	0.90 (0.34-2.26)	-	-	-	-
Received TB treatment	0.40 (0.15-0.95)	0.17 (0.05-0.49)	-	-	-	-
Diagnosis is malaria	0.04 (0.00-0.47)	-	0.07 (0.00-1.19)	-	-	-
Received malaria treatment	0.06 (0.00-0.86)	-	0.20 (0.00-5.66)	-	-	-
Diagnosis is invasive fungal disease	5.90 (1.00-41.00)	-	-	3.37 (0.41-31.43)	-	-
Received antifungal	1.78 (0.66-4.38)	-	-	0.79 (0.26-2.23)	-	-
Time to antibacterial therapy (per hour)	1.01 (0.98-1.03)	-	-	-	1.02 (0.99-1.04)	-
Vol of IV fluid (L)	1.19 (0.79-1.800	-	-	-	-	0.52 (0.29-0.91)
