# Supplementary material for: A Longitudinal, Observational Study of Etiology and Long-Term Outcomes of Sepsis in Malawi Revealing the Key Role of Disseminated Tuberculosis
Source: Clin Infect Dis. 2021 Aug 18;74(10):1840–9. doi: 10.1093/cid/ciab710 (PMC9155594; doi:10.1093/cid/ciab710)
Supplement: ciab710_suppl_Supplementary_Methods [file ciab710_suppl_supplementary_methods.pdf]

A longitudinal observational study of aetiology and long-term outcomes of sepsis in Malawi revealing the key role of disseminated tuberculosis

SUPPLEMENTARY METHODS

Diagnostic testing

Bedside capillary blood testing

At baseline, blood was tested for HIV-1/2 antibodies using a Determine 1/2 rapid diagnostic test (RDT) (Abbott Laboratories, USA) with positive results confirmed with a second HIV 1/2 RDT (UniGold HIV 1/2 Trinity Biotech Inc., Ireland) as per Malawian national guidance, if patients consented.

Bedside testing of capillary blood for lactate (Lactate Pro 2, Arkay, Japan) and P. falciparum HRP-2 antigen (Paracheck Pf, Orchid Biomedical, India) was carried out for all participants.

Laboratory testing

Venous blood was collected aseptically and transported to the Malawi-Liverpool-Wellcome (MLW) clinical research laboratories for standard haematologic (Beckman Coulter HmX Haematology Analyser, Beckman Coulter, USA) and biochemical (Beckman Coulter AU480 Chemistry Analyser, Beckman Coulter, USA) analyses: full blood count and creatinine, urea and electrolytes, and CD4 cell count quantification (Becton Dickinson FACSCount, Becton Dickinson, USA).

One aerobic blood culture was inoculated with 5-10ml of blood and incubated using an automated system (BacT/Alert, BioMerieux) in the MLW labs and cultured isolates identified to genus level (all) and species level using the API system and standard techniques[1]. Antimicrobial sensitivity testing was undertaken using the disc diffusion method following British Society for Antimicrobial Chemotherapy (BSAC) guidelines. Cefpodoxime resistance was used as a proxy for production of extended-spectrum beta-lactamase enzymes. Coagulase-negative Staphylococci*, Bacillus spp.,* diptheroids and alpha-haemolytic Streptococci other than *S. pneumoniae* were considered as contaminants. Acute and convalescent blood samples and urine samples were stored at -80°C for further testing.

Testing for disseminated TB was only carried out in those who were known to be HIV infected or of unknown HIV status. One mycobacterial blood culture (BACTEC Myco/F Lytic, Becton Dickinson, USA) was inoculated with 5-7ml of blood and cultured at 37°C in the TB laboratory of the Malawi College of medicine. It was inspected daily for the first 14 days and then every two days thereafter for fluorescence with a handheld Wood's lamp. On the detection of fluorescence the contents were centrifuged and examined by Gram's and ZN stain to exclude contamination, and inoculated into mycobacterial growth indicator tubes (MGIT, Beckton Dickinson, USA) for up to 6 weeks. Subsequently cultured isolates were classified as *M. tuberculosis* or non-tuberculous Mycobacteria by microscopic cording and MPT-64 lateral flow assays (TAUNS Laboratories, Japan). After 8 weeks if no fluorescence was seen then centrifugation and microscopy was carried out; if no organisms were seen then this was classed as "no growth".

Batched urinary lipoarabinomannan testing using lateral flow assays (Alere determine TB, Abbot Laboratories, USA) was carried out on thawed frozen (-80°C) urine as per the manufacturer's instructions. Urine was thawed, briefly centrifuged to remove sediment and 60 microlitres of urine was applied to the sample pad and the result read after 25 minutes by comparing to the provided reference scale card. If a line was visible in the patient window of the test and darker than the lightest positive line on the reference card then it was considered positive. If a line was visible but lighter than the lightest positive line on the reference card, or if no line was visible, then it was considered negative. If no line was visible in the control window of the test then the test was considered invalid, and repeated. The results were read independently by two readers, who were unaware of the other reader's finding. A tie-break read by a third reader who was unaware of the findings of the other two readers was undertaken in the event of disagreement.

Testing of sputum for tuberculosis using Xpert MTB/RIF and microscopy and culture of CSF were carried out when there was suspicion of pulmonary tuberculosis or meningitis, respectively, by the clinical team. Sputum Xpert MTB/RIF testing (Cepheid, United States) was carried out as per the manufacturer's instructions in the QECH hospital laboratories: sputum specimens were mixed with sample reagent and incubated at room temperature for 15 minutes. The liquefied specimen was then loaded into the Xpert MTB/RIF test cartridge for processing. CSF culture was carried out at the MLW laboratories: CSF was cultured on blood, chocolate and Sabouraud agar and then identification of any growth undertaken as for blood cultures above. In addition CSF was tested for cryptococcal antigen by lateral flow assay (Bruker Corporation, USA) as per the manufacturer's instructions.

Stored frozen (-80°C) sample sets of acute and convalescent sera were shipped on dry ice to the Public Health England Rare and Imported Pathogens Laboratory, Porton Down, UK for testing of convalescent sera for IgG and IgM to Chikungunya and Dengue by enzyme immunoassay (EIA), spotted fever group and epidemic typhus group Rickettsioses by immunofluorescence assay (IFA) and IgM to Leptospira by EIA. All available convalescent sera (n=149) underwent serologic testing as well as a sample of acute sera (n = 41, 33 of which had no available paired convalescent serum).

A random sample of stored frozen acute sera were also tested on a multiplex TaqMan Array card (Thermo Fisher Scientific, United, States) established by Public Health England to look for 46 bacterial and viral pathogens (See Supplementary Method Table 1). 122 serum samples were taken forward for testing; these were selected from all available acute sera by random sampling weighted 10:1 in favour of samples in which no diagnosis had been made by culture, urinary LAM testing, or sputum Xpert MTB/RIF. Samples were processed within a containment level 3 laboratory in preparation for sample extraction. Once processed, selected samples were then extracted on a MagNA Pure 96 instrument, stored in 2ml microtubes and frozen until tested. Samples were then processed on TaqMan Array cards within a containment level 2 laboratory using a defined working method. All positive hits were recorded on a master result sheet, including specific card internal controls. Extraction controls were also processed and recorded.

Channels 1, 3, 5, 7 (Bacterial panel)		Channels 2, 4, 6, 8 (Viral panel)	
Port		Port	
Left	Right	Left	Right
1 RNase P (control)	25 Staph aureus MecA	1 RNase P (control)	25 Lassa virus Pineo
2 18S RNA (control)	26 Proteus sp.	2 MS2 control	26 Lassa virus Weller
3 MS2 (control)	27. Neisseria meningitidis	3 Pan influenza A	27. Chikungunya virus
4 Streptococcus pneumoniae#1	28. Pseudomonas aeruginosa#1	4 Pan influenza B	28. Yellow fever virus
5. Haemophilus influenza#1	29. Chlamydia pneumoniae	5. Respiratory syncytial virus A	29. Ebola Bundibugyo virus
6. Bordetella pertussis ptx	30. Mycoplasma pneumoniae#1	6. Respiratory syncytial virus B	30. Ebola Zaire virus
7. Bordetella pertussis IS481	31. Brucella sp.	7. Measles virus	31. Ebola Sudan virus
8. Coxiella burnetii	32. Pan-Borrelia	8. Mumps virus	32. Ebola Cote D'ivoire virus
9. Klebsiella pneumoniae#1	33. Bordetella pertussis IS481#2	9. Rubella virus	33. Pan Ebola virus
10. Vibrio cholera	34. EAE (intimin gene) E. coli	10. Coronavirus GP2 (OC43/HKU1)	34. Pan Marburg virus
11. Shigella sp.	35. Pseudomonas aeruginosa #2	11. Coronavirus 229E	35. Marburg virus#1
12. Salmonella hilA	36. Enterobacteriaceae (generic)	12. Coronavirus NL63	36. Marburg virus #2
13. Salmonella ttr	37. Streptococcus pneumoniae#2	13. Coronavirus MERS	37. West Nile virus
14. Campylobacter jejuni/coli	38. Klebsiella pneumoniae#2	14. Rotavirus	38. Rift Valley Fever virus
15. Enteroagluttigenic E. coli	39. Streptococcus pyogenes#2	15. Norovirus GII	39. Varicella zoster virus#2
16. E. coli generic #1	40. E. coli generic#2	16. Dengue virus#1	40. Enteroviruses
17. Verotoxin 1	41. Haemophilus influenza#2	17. Dengue virus#2	41. Adenovirus #1
18. Verotoxin 2	42. Mycoplasma pneumoniae#2	18. Lassa virus Jo-SL	42. Adenovirus #2
19. Leptospira sp.	43. Streptococcus agalactiae #1	19. Lassa virus Josiah	43. Human metapneumovirus
20. Rickettsia sp.	44. Streptococcus agalactiae#2	20. Lassa virus Liberia	44. Epstein-Barr virus #1
21. Streptococcus pyogenes#1	45. Enterobacter cloacae#1 DNAJ	21. Lassa virus Macenta	45. Rhinovirus #2
22. Pan-streptococcus #1	46. Enterobacter cloacae#2 Rpo2	22. Lassa virus Nigeria 1	46. Cytomegalovirus #2 br
23. Pan-streptococcus #2	47. CMV#1	23. Lassa virus Nigeria 2	47. Rhinovirus #1
24. S aureus	48. H5 Flu	24. Lassa virus Nigeria 4	48. Free

Supplementary Methods Table 1: TaqMan array card layout for the multipathogen panel. The microfluidic cards contain 384 individual wells arranged in 8 channels with 48 wells per channel. The design of the multipathogen array card was maximised to contain primer sets to detect 50 individual bacterial and viral pathogens. Channels 1, 3, 5 and 7 contain mainly primers against bacterial targets and channels 2, 4, 6 and 8 contain primers against viral targets. Each channel also contains RNAse P and MS2 internal controls. For several pathogens, more than one genetic target is included (indicated by #

Statistical Analysis

Sample size

The study was powered to detect clinically relevant risk factors for death; we made the a priori assumption that a risk ratio of 2 or more is clinically relevant in this setting and patient population. We initially aimed to recruit 250 patients; with this number of patients and assuming a case fatality ratio of 50% (as seen in previous studies in Malawi) would have 80% power to detect risk factors for death with prevalence of 20-50% that confer a risk ratio (RR) of 1.5-3; therefore, a RR of 2 is likely to be detected. Resource considerations during the study resulted in a reduction of a feasible sample size to 225; repeating a power calculation with this number of participants suggested 80% power to detect risk factors conferring a RR of 2 with 25-50% prevalence, assuming 50% mortality.

Summary statistics

Continuous values were summarized by medians and interquartile ranges, categorical variables by proportions and exact binomial confidence intervals. Differences between groups were assessed with bootstrapped differences in median or proportion with 95% confidence intervals constructed using the bias-corrected and accelerated bootstrap interval[2]. Where carried out, tests of difference in proportions between two groups use Fisher's exact test.

Estimating proportion of infections sensitive to ceftriaxone

To estimate the proportion of microbiologically infections that would be sensitive to ceftriaxone we used the actual antimicrobial sensitivity testing if that were available (for aerobic culture isolates); if not, we assumed that *Streptococcus pneumoniae*, *Salmonella* spp., Enterobacterales, *Acinetobacter* spp. were susceptible to ceftriaxone and *Pseudomonas* spp. and *Enterococcus* spp. were nonsusceptible. In addition, regardless of antimicrobial sensitivity testing result we assumed that organisms with an inducible chromosomal AmpC (*Enterobacter, Serratia, Citrobacter, Aeromonas, Provadencia, Morganella* spp.) were nonsusceptible to ceftriaxone. We assumed that *Leptospira spp.* were susceptible to ceftriaxone.

Estimating effects of treatments on outcome

We aimed to identify the effect of treatment on death by 28 days. We first hypothesized a causal structure (Supplementary Figure E1) of host variables (e.g. HIV, CD4 count, haemoglobin), infection (e.g. causative pathogen) and sepsis severity (e.g. lactate, blood pressure). We then used the R package *dagitty*[3] to identify that, to identify the causal effect of treatment variables on outcome, we need to condition on host, infection and severity variables, conditional on the hypothesized causal structure being correct. To account for collinearity of severity and host variables and to undertake dimensionality reduction on the large number of putative variables we used principal components analysis after log-transforming heavily skewed variables (Supplementary Table E1), scaling continuous variables to mean 0 and standard deviation 1 and coding binary variables as {0,1}. We initially included 20 a priori selected variables, and assessed correlation with Pearson correlation

coefficients and excluded those that were very strongly correlated (coefficient > 0.8, Supplementary Figure E8).

To manage the problem of separation (where some variables perfectly predict outcome and so maximum likelihood-fit parameter estimates become unidentifiable) we fit the models in a Bayesian framework with student's t-distribution with 4 degrees of freedom and a scale parameter of 2.5 as priors for all parameters, following Gelman et al[4]. Convergence was assessed by inspection of traceplots and by the Gelman-Rubin convergence diagnostic ($\hat{R}$) being less than 1.1. Missing data (which were unusual, Supplementary Figure E10) were imputed by chained equations using the *mice* v3.11.0 package in R[5]. Ten imputed datasets were generated, models were fit on each dataset using *brm_multiple()* then the posterior estimates from all chains were pooled to generate summary statistics. Models were fit with Stan v2.21.0 via the R *brms* v2.13.5 package[6,7], with 4 chains per dataset each with 2000 iterations in total, with 1000 warm up iterations.

We first constructed models with between one and five of the host-severity variable principal components as predictors of 28-day mortality and used approximate (pareto -smoothed importance sampling) leave-one out cross validation as implemented in the *loo* package in R[8] to identify the model with the best out of sample prediction (quantified by the expected log pointwise predictive value, ELPD). This model became the base model, and we assessed the effect of different therapies by adding, in turn, a binary variable indicating receipt of different classes of antimicrobials (antituberculous agents, antifungals, antimalarials) and, in each case, a binary variable indicating the presence of absence of the corresponding diagnosis

(TB, invasive fungal disease, and malaria). We also included time-to-antibacterial therapy (defined as the time from ED registration to receipt of any antibacterial) as a continuous variable both as a linear variable and represented with restricted cubic splines with 3 knots at the centiles {0.1,0.5,0.9}; volume of intravenous fluid received was also modelled with the same spline function. Both variables were mean centred and scaled by their standard deviation before being included in the models.

The outputs from the models are presented as odds ratios (OR) with a point estimate (the posterior median) with 95% credible intervals (CrI), except for the nonlinear models which are presented as the marginal effect of the predictor on the probability of outcome with 95% credible intervals. Presence or absence of arboviral infections was not included in any mortality models as diagnostics testing was largely done only on convalescent samples.

REFERENCES

1 Barrow GI, Cowan ST, Steel KJ, Feltham RKA. Cowan and Steel's manual for the identification of medical bacteria. Cambridge University Press, 1993.

2 Efron B. Better bootstrap confidence intervals. *J Am Stat Assoc* 1987; **82**: 171–85.

3 Textor J, van der Zander B, Gilthorpe MS, Liśkiewicz M, Ellison GTH. Robust causal inference using directed acyclic graphs: the R package 'dagitty'. *Int J Epidemiol* 2017; **45**: dyw341.

4 Gelman A, Carlin JB, Stern HS, Dunson DB, Vehtari A, Rubin DB. Bayesian data analysis, 3rd edn. Chapman and Hall/CRC, 2004.

5 Buuren S van, Groothuis-Oudshoorn K. mice : Multivariate Imputation by

Chained Equations in R. *J Stat Softw* 2011; **45**: 1–67.

6 Carpenter B, Gelman A, Hoffman MD, *et al.* Stan: A Probabilistic Programming Language. *J Stat Softw* 2017; **76**: 1–32.

7 Bürkner P-C. brms: An R Package for Bayesian Multilevel Models Using Stan. *J Stat Softw* 2017; **80**: 1–28.

8 Vehtari A, Gelman A, Gabry J. Practical Bayesian model evaluation using leave-one-out cross-validation and WAIC. *Stat Comput* 2017; **27**: 1413–32.
