# Supplementary material for: A Longitudinal, Observational Study of Etiology and Long-Term Outcomes of Sepsis in Malawi Revealing the Key Role of Disseminated Tuberculosis
Source: Clin Infect Dis. 2021 Aug 18;74(10):1840–9. doi: 10.1093/cid/ciab710 (PMC9155594; doi:10.1093/cid/ciab710)
Supplement: ciab710_suppl_Supplementary_Material [file ciab710_suppl_supplementary_material.docx]

STROBE cohort study checklist:

**Aetiology and determinants of outcome in sepsis in an urban African centre: an observational cohort studY**

| Description | Item number | Recommendation | Present? | Where described |
| --- | --- | --- | --- | --- |
| Title and abstract | 1 | (*a*) Indicate the study’s design with a commonly used term in the title or the abstract | Yes | Title |
|  |  | (*b*) Provide in the abstract an informative and balanced summary of what was done and what was found | Yes | Abstract |
| Introduction |  |  |  |  |
| Background/rationale | 2 | Explain the scientific background and rationale for the investigation being reported |  | Introduction |
| Objectives | 3 | State specific objectives, including any prespecified hypotheses |  | Introduction |
| Methods |  |  |  |  |
| Study design | 4 | Present key elements of study design early in the paper | Yes | Methods/supp. methods |
| Setting | 5 | Describe the setting, locations, and relevant dates, including periods of recruitment, exposure, follow-up, and data collection | Yes | Methods |
| Participants | 6 | (*a*) Give the eligibility criteria, and the sources and methods of selection of participants. Describe methods of follow-up | Yes | Methods |
|  |  | (*b*) For matched studies, give matching criteria and number of exposed and unexposed | NA | NA |
| Variables | 7 | Clearly define all outcomes, exposures, predictors, potential confounders, and effect modifiers. Give diagnostic criteria, if applicable | Yes | Methods/supp. methods |
| Data sources/ measurement | 8 | For each variable of interest, give sources of data and details of methods of assessment (measurement). Describe comparability of assessment methods if there is more than one group | Yes | Methods/ supp. methods |
| Bias | 9 | Describe any efforts to address potential sources of bias | Yes | Methods/ supp. methods |
| Study size | 10 | Explain how the study size was arrived at | Yes | Supp methods |
| Quantitative variables | 11 | Explain how quantitative variables were handled in the analyses. If applicable, describe which groupings were chosen and why | Yes | Methods/supp. methods |
| Results |  |  |  |  |
| Participants | 13 | (a) Report numbers of individuals at each stage of study—eg numbers potentially eligible, examined for eligibility, confirmed eligible, included in the study, completing follow-up, and analysed | Yes | Supp. Figure 2 |
|  |  | (b) Give reasons for non-participation at each stage | Yes | Supp. Figure 2 |
|  |  | (c) Consider use of a flow diagram | Yes | Supp. Figure 2 |
| Descriptive data | 14 | (a) Give characteristics of study participants (eg demographic, clinical, social) and information on exposures and potential confounders | Yes | Results |
|  |  | (b) Indicate number of participants with missing data for each variable of interest | Yes | Supp. Figure 10 |
|  |  | (c) Summarise follow-up time (eg, average and total amount) | Yes | Results |
| Outcome data | 15 | Report numbers of outcome events or summary measures over time | Yes | Results |
| Main results | 16 | (*a*) Give unadjusted estimates and, if applicable, confounder-adjusted estimates and their precision (eg, 95% confidence interval). Make clear which confounders were adjusted for and why they were included | Yes | Results |
|  |  | (*b*) Report category boundaries when continuous variables were categorized | NA | NA |
|  |  | (*c*) If relevant, consider translating estimates of relative risk into absolute risk for a meaningful time period | NA | NA |
| Other analyses | 17 | Report other analyses done—eg analyses of subgroups and interactions, and sensitivity analyses | NA | NA |
| Discussion |  |  |  |  |
| Key results | 18 | Summarise key results with reference to study objectives | Yes | Discussion |
| Limitations | 19 | Discuss limitations of the study, taking into account sources of potential bias or imprecision. Discuss both direction and magnitude of any potential bias | Yes | Discussion |
| Interpretation | 20 | Give a cautious overall interpretation of results considering objectives, limitations, multiplicity of analyses, results from similar studies, and other relevant evidence | Yes | Discussion |
| Generalisability | 21 | Discuss the generalisability (external validity) of the study results | Yes | Discussion |
| Other information |  |  |  |  |
| Funding | 22 | Give the source of funding and the role of the funders for the present study and, if applicable, for the original study on which the present article is based | Yes | Funding |
